# Dissociating sensory from decision processes in human perceptual decision making

**DOI:** 10.1038/srep18253

**Published:** 2015-12-15

**Authors:** Pim Mostert, Peter Kok, Floris P. de Lange

**Affiliations:** 1 Radboud University, Donders Institute for Brain, Cognition and Behaviour, 6500 HB, Nijmegen, The Netherlands

## Abstract

A key question within systems neuroscience is how the brain translates physical stimulation into a behavioral response: perceptual decision making. To answer this question, it is important to dissociate the neural activity underlying the encoding of sensory information from the activity underlying the subsequent temporal integration into a decision variable. Here, we adopted a decoding approach to empirically assess this dissociation in human magnetoencephalography recordings. We used a functional localizer to identify the neural signature that reflects sensory-specific processes, and subsequently traced this signature while subjects were engaged in a perceptual decision making task. Our results revealed a temporal dissociation in which sensory processing was limited to an early time window and consistent with occipital areas, whereas decision-related processing became increasingly pronounced over time, and involved parietal and frontal areas. We found that the sensory processing accurately reflected the physical stimulus, irrespective of the eventual decision. Moreover, the sensory representation was stable and maintained over time when it was required for a subsequent decision, but unstable and variable over time when it was task-irrelevant. In contrast, decision-related activity displayed long-lasting sustained components. Together, our approach dissects neuro-anatomically and functionally distinct contributions to perceptual decisions.

A substantial part of cognitive neuroscience is devoted to the question of how the brain translates physical stimulation into behavioral decisions - an operation known as perceptual decision making^1^,^2^. Theoretical frameworks posit that perceptual decisions arise from a sequence of functionally distinct processes^3^. These frameworks distinguish the sensory process, where the physical stimulus is encoded into internal sensory evidence, from the decision process, that integrates this sensory evidence over time into a decision variable. A number of studies have revealed electrophysiological markers of these processes in humans, using a variety of paradigms^4^,^5^,^6^,^7^,^8^,^9^,^10^. Here we focus on the simplest of perceptual decision making tasks, stimulus detection, in which subjects are required to report the presence or absence of a stimulus in noise, and aimed to dissociate the neural activity underlying the sensory process from that underlying the decision process.

In this type of task, the behavioral response is typically used to post-hoc sort the data into the four stimulus/response-categories [hits, correct rejects (CRs), misses and false alarms (FAs)] in order to separate the underlying sensory and decision processes^11^. By contrasting categories that differ on one dimension only (stimulus presence or behavioral report), one would expect to obtain the neural activity underlying the process that corresponds to that factor (e.g. refs 12, 13, 14, 15, 16). This approach suffers however from at least two conceptual problems.

First, the sensory and decision process are not fully dissociated. For example, if a stimulus is erroneously encoded during the sensory process, then an incorrect decision will likely follow. Thus the response factor targets not only the decision process, but also the sensory process. Moreover, the stimulus factor may target not only differences in the sensory process, but also the decision process. This is because, even when the final behavioral outcome is equal, the temporal integration during the decision process likely follows a deviating trajectory for incorrect decisions as compared to correct ones^3^,^17^.

Second, as the post-hoc defined response factor is an observed variable, it is not under the experimental control of the researcher. As a result, any relation between response and neural activity may be confounded by third variables. For instance, perceptual decisions are modulated by ongoing fluctuations in neural activity or attention^18^,^19^,^20^,^21^,^22^. Thus, the response factor may target other processes, such as attention, that may subsequently modulate the sensory or decision process. Therefore, it is often unclear how to interpret differential neural activity revealed by this factor.

In summary, differential neural activity obtained by comparing the stimulus/response-categories is difficult to interpret, and conclusions derived from this approach require caution. Here, we adopted a different approach, which does not suffer from these limitations, to dissociate sensory from decision processes in human magnetoencephalography (MEG) recordings. We first identified, using a separate localizer task, the neural activity corresponding to the sensory process in absence of a decision. Then, we traced the neural signature of the sensory process while subjects performed a perceptual decision making task. We found that this between-task generalization method reliably identified the sensory representation in an early time window. Moreover, we observed that this sensory representation was stabilized and maintained over time, but only when required for a decision. In summary, our approach yields a new window onto the role of sensory processes during perceptual decision making.

## Materials and Methods

### Subjects

Twenty-four healthy human volunteers, recruited from the institute’s subject pool, participated in the experiment and received either monetary compensation or study credits. The study was approved by the local ethics committee (CMO Arnhem-Nijmegen, Radboud University Medical Center) under the general ethics approval (“Imaging Human Cognition”, CMO 2014/288), and the experiment was conducted in compliance with these guidelines. Written informed consent was obtained from each individual. Two subjects were excluded during preprocessing due to insufficient data quality (severe eye and muscle artifacts). The remaining twenty-two subjects (nine females, age 19–30 years) had normal or corrected-to-normal vision.

### Stimuli

**Figure 1:**
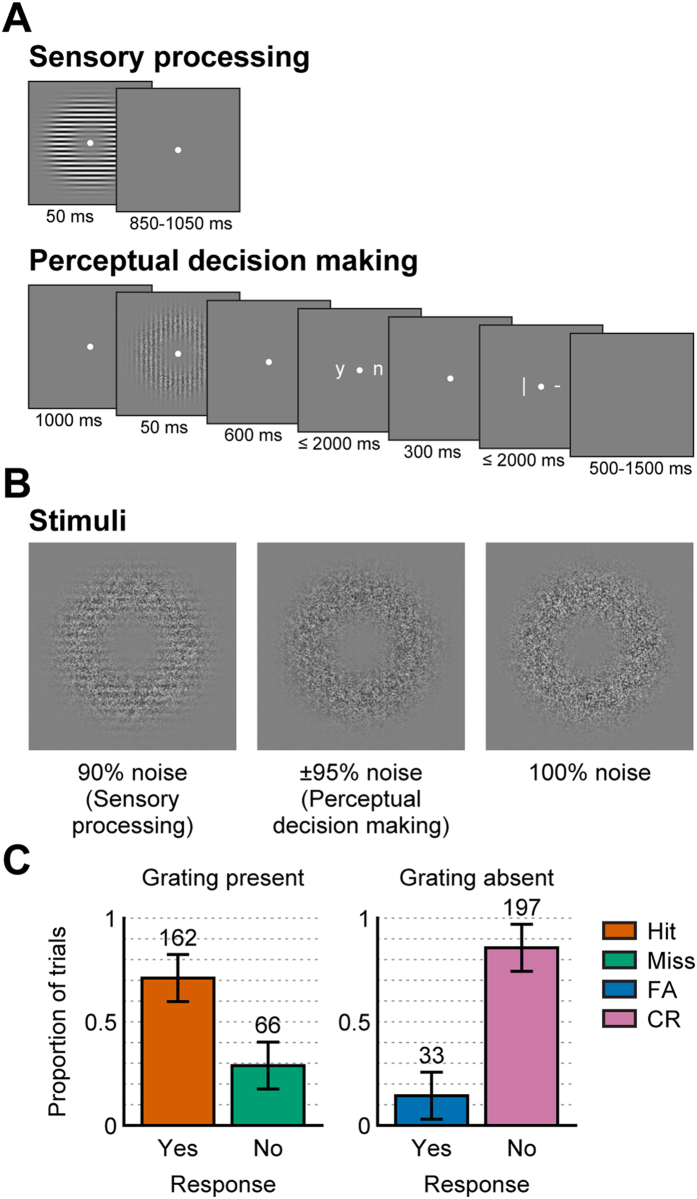
Experimental paradigm and behavioral results. (**A**) In the sensory processing blocks, the noise/grating stimuli were irrelevant and unattended. In the perceptual decision making blocks, a decision had to be made regarding the presence or absence of a grating. Grating visibility is enhanced for illustrative purposes. (*B*) Example stimuli. The noise level in perceptual decision making blocks was tailored to individual detection thresholds. (*C*) Average response proportions in perceptual decision making blocks, for grating present and grating absent trials, color-coded according to the four stimulus/response-categories. The numbers denote the average number of trials available in each of these categories. Error bars depict standard deviations.

Stimulation consisted of visual noise presented on a gray (50% of maximum pixel intensity, luminance: 321 cd/m^2^) background, which could contain an embedded horizontal or vertical grating (Fig. 1B). Noise patches consisted of white noise that was subsequently smoothed with a Gaussian kernel (SD = 0.05°). Gratings were sine waves with a spatial frequency of 1 cycle/° and random phase. The gratings were embedded in the noise by averaging the two images, weighted according to a desired noise level (0%: full-contrast noise-free grating; 100%: pure noise). The pixel values of the resulting image were rescaled such that the minimum and maximum values mapped onto 0% and 100% of maximum pixel intensity, respectively. Finally, to obtain an annulus, all stimuli were masked with a radially oriented Gaussian mask (SD = 2°) centered at a radius of 6°, resulting in an overall diameter of approximately 24°. Stimuli were generated and presented using MATLAB (The Mathworks, Inc., Natick, Massachusetts, United States) and the Psychophysics Toolbox extensions^23^.

### Procedure and experimental design

Each subject participated in a behavioral practice session in order to become familiar with the experiment. The practice sessions were scheduled at most two days before the main experimental session. The main session involved three different types of blocks. After an initial staircase block (see below), subjects performed six sensory processing (localizer) blocks and six perceptual decision making blocks in alternating order, starting with a sensory processing block.

In the staircase block, we used the Quest staircase procedure^24^ (as implemented in PsychToolbox) to estimate the individual noise level at which each subject correctly detects a grating embedded in noise in 70% of the cases. The procedure was similar to the perceptual decision making blocks (see below), except that subjects only had to indicate the presence or absence of a grating and not its orientation. In addition, subjects received feedback on every trial. Only the trials in which a grating was actually presented were used to update the staircase. For the last eighteen subjects, convergence of the staircase was visually inspected at the end of the block and, if convergence was not yet achieved, more staircase trials were administered.

Each sensory processing block comprised 120 trials during which subjects were presented with a brief stimulus for 50 ms (Fig. 1A). In 50% of the trials, the stimulus was pure noise (referred to as noise trials) whereas the other 50% contained a grating embedded in the noise (referred to as grating trials). In half of the grating trials, the grating had a horizontal orientation whereas a vertical grating was present in the other half. The noise level of the grating trials was set to 90%. This value was chosen such that it was sufficiently high to be comparable to the stimuli presented during the perceptual decision making blocks, yet low enough to ensure clear visibility of the gratings (Fig. 1B). For the first seven subjects, the inter-trial interval was fixed to 950 ms, whereas for the remaining fifteen subjects this interval was randomly drawn from a uniform distribution between 850 and 1050 ms. As we were specifically interested in the neural signature of sensory processing in the absence of higher-level attentional and/or decision processes, subjects were required to perform a task at fixation to draw attention away from the stimuli. In 10% of the trials, and balanced across stimulus conditions, the fixation dot (diameter 0.2°) was absent during the 50 ms stimulus presentation. Subjects were instructed to report such a “blink” by pressing a button as quickly as possible. These “oddball” trials, as well as the non-oddball trials on which subjects erroneously pressed a button, were excluded from further analyses.

A perceptual decision making block comprised 80 trials. Each trial began with a 1000 ms fixation period, after which a stimulus was briefly presented for 50 ms (Fig. 1A). Again, this stimulus was either pure noise (in 50% of trials), or contained a grating (vertical grating in 25% and horizontal grating in 25% of the trials). Unlike the sensory processing blocks, the noise level was set to a much higher level, namely the individual threshold of perception as determined by the staircase procedure. Then, after 600 ms the letters ‘y’ and ‘n’ (as abbreviations for “yes” and “no”, respectively) were displayed, centered around the fixation dot. Subjects reported their decision as to whether they had perceived a grating or not by pressing a button with either the left or the right hand, corresponding to the position of the letter that matches their decision. The position of the letters (‘y’ left and ‘n’ right, or ‘n’ left and ‘y’ right) was randomized across trials to orthogonalize perceptual decision and motor response preparation. Trials in which no button press was made were discarded from further analysis. After button press, or after 2000 ms in case of no button press, another fixation period of 300 ms followed, after which a second display with a horizontal and a vertical line was presented to inquire the subject’s decision regarding the orientation of the grating. The position of the lines were also randomized across trials. Subjects were instructed to, in trials where they perceived only noise, indicate the orientation to which they thought the noise was most similar. Finally, a blank screen was presented with an inter-trial interval drawn randomly from a uniform distribution between 500 and 1500 ms

The two different grating orientations were included in the design because we had hypotheses regarding not only the sensory processing of stimulus presence versus absence, but also regarding the processing of orientation-specific signals. However, since we were not able to successfully decode the orientation of stimuli from the MEG signal, stimulus orientation was left out of further consideration.

### Behavioral analysis

The observer’s sensitivity *d’* and criterion *c* were calculated as follows:


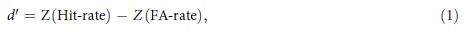



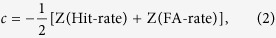


where Z(…) is the inverse standard normal distribution.

### MEG recording and preprocessing

Whole-head neural recordings were obtained using a 275-channel MEG system with axial gradiometers (VSM/CTF Systems, Coquitlam, BC, Canada) located in a magnetically shielded room. Throughout the experiment, head position was monitored online, and corrected if necessary, using three fiducial coils that were placed on the nasion and on earplugs in both ears. Behavioral responses were made using two MEG-compatible button boxes, one for each hand. Visual stimulation was projected from outside the magnetically shielded room, via mirrors onto a screen in front of the subject. Furthermore, both horizontal and vertical electrooculograms (EOGs), as well as an electrocardiogram (ECG) were recorded to facilitate removal of eye- and heart-related artifacts. All signals were sampled at a rate of 1200 Hz.

The data were preprocessed offline using FieldTrip^25^ (www.fieldtriptoolbox.org). Notch filters were applied at 50, 100 and 150 Hz to remove line noise and its harmonics. In order to identify artifacts, the variance (collapsed over channels and time) was calculated for each trial. Trials with large variances were subsequently selected for manual inspection and removed if they contained excessive and irregular artifacts. Independent component analysis was subsequently used to remove regular artifacts, such as heartbeats and eye blinks. Specifically, for each subject, the independent components were correlated to both EOGs and the ECG to identify potentially contaminating components, and these were subsequently inspected manually before removal. For covariance computation in the source localization, the remaining components were transformed back to sensor-space and subsequently low-pass filtered at a cut-off frequency of 30 Hz. For the decoding analysis however, the components were kept in component-space to ensure that its covariance matrix is of full rank as required for this analysis (see below). Finally, the data were baseline corrected on the interval of −200 to 0 ms relative to stimulus onset.

### Decoding analysis

The general idea behind a neural decoding analysis is that it attempts to invert the encoding process. The encoding process determines the neural responses as a function of some parameter, for instance a physical stimulus or an experimental condition. A decoding analysis aims to invert this function in order to unveil the encoded parameter as a function of neural signals. The first step in achieving this is to estimate a forward model that describes the encoding process. For this, a data set is used in which the parameter is known and the corresponding neural signals are recorded empirically. Secondly, an inverse model is estimated on the basis of the forward model and subject to some criterion of optimality. We will refer to such an inverse model as a decoder, as it takes neural signals as input and produces an estimate of the encoded parameter as output.

Our method is largely based on linear discriminant analysis as described in ref. 26. Linear discriminant analysis attempts to find a linear transformation of the data, such that the resulting signal is optimally discriminative between two classes. First, we demeaned the data such that for each time point and for each feature, the average over trials equals zero. Features here refer to independent components (see *MEG recording and preprocessing*). Let 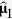 and 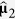 be column vectors of length *F*, where *F* is the number of features, that contain the neural responses in the training set for class 1 and 2, respectively, at some time point and averaged across trials. Then, the weights vector **w** that optimally discriminates between classes on the basis of the features is given by^26^:


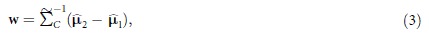


where 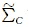 is the common regularized covariance matrix (see below). Next, let **X** be a matrix of size *F* × *N*, where *N* refers to the number of trials, that contains the data to be decoded. The decoded signal **y** is then obtained by:


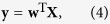


where (…)^T^ denotes the matrix transpose. In linear discriminant analysis, this signal is transformed into a discrete class membership by specifying a cut-off value for **y**. Here, we deviated from standard linear discriminant analysis. Rather than assigning a discrete label to a trial, we were interested in a continuous measure of the degree to which a class is encoded in the neural signals. Thus, we did not apply a binary cut-off to the decoded signal. Furthermore, to make this signal comparable across time points, we added a normalization factor to the weights vector such that the mean difference in the decoded signal between classes equals a value of one. This is accomplished by modifying equation (3) into:


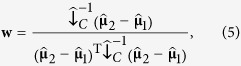


in which the denominator is the normalization factor. Thus, equation (4) and (5) constitute our final decoder. We term the output of the decoder the “discriminant channel”, as it optimally discriminates between the two classes that it was trained on. Specifically, if we denote the mean discriminant channel amplitude in class 1 and 2 by 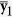 and 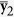, respectively, then we expect 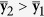 if there is information in the neural signals pertaining to the classes, whereas we expect 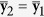 if no such information is available. In other words, the difference in the discriminant channel between classes is a measure of the discriminability of these classes on the basis of the neural signals.

The interpretation of the decoder’s output as a discriminant channel is further motivated by drawing an analogy to linearly constrained minimum variance (LCMV) spatial filters^27^, more commonly known as beamformers. A beamformer is an inverse model to estimate neural activity at the source level, given sensor-level activity. Beamformers are commonly used to extract time courses of activity of some source-level region of interest into so-called virtual channels, as if the activity in that region had been recorded directly. The forward model in this method, also known as the leadfield, describes how sensor-level activity varies as a function of source-level activity. The analogy is made by noting that equation (5) is equivalent to the calculation of beamformers, but whereas the forward models for beamformers are defined in a spatial sense, the forward model in our decoding method is defined in a discriminatory sense. It describes how activity at the sensor-level varies as a function of a discriminating parameter, namely the class, and is in fact the difference event-related field 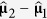. If this difference event-related field is entered in the LCMV beamformer formula^27^ as the forward model, then equation (5) is the result. Thus, the output of our decoder is analogous to a virtual channel, but defined in a discriminatory rather than a spatial sense, hence the name discriminant channel. Contrary to spatially defined virtual channels, the discriminant channel may stem from a wide array of neural sources, and its output is the collective activity of this array. Finally, we point out that the normalization in equation (5) corresponds directly to the unit-gain constraint in LCMV beamformers^27^.

To facilitate comparison to other decoding methods, we included the results in the Supplementary Information normalized by their standard deviation at the individual level, yielding grand averaged Cohen’s d effect sizes. Moreover, we also carried out an additional analysis in which we did set a cut-off value in order to assign a discrete class to each trial. This value was chosen as 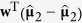 , because this results in equal type I and type II error rates^28^. By assigning discrete labels to trials, the results can be expressed as the proportion of trials that are assigned to either of two classes.

An important element in the decoding analysis is that it takes advantage of correlations between features in order to suppress noise. Let the column vector **x**_*i*_ of length *F* denote the data in trial *i*, and define the corresponding mean as:


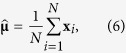


then the estimated covariance matrix is obtained by:


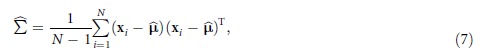


For optimal noise suppression, we improved this estimation by means of regularization by shrinkage, using the analytically determined optimal shrinkage parameter (for details, see ref. 26). This procedure was performed separately within each condition, and the resulting condition-specific regularized covariance matrices were subsequently averaged to obtain the common regularized covariance matrix 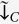.

The decoding analysis outlined above was performed in a time-resolved manner by applying it sequentially at each time point, in steps of 5 ms, resulting in an array of decoders. To improve the signal-to-noise ratio, the data were first averaged within a window of 29.2 ms centered around the time point of interest. The window length of 29.2 ms is based on an *a priori* chosen length of 30 ms, but minus one sample such that the window contained an odd number of samples for symmetric centering. Thus, the output of the time-resolved analysis is a one-dimensional time series of the amplitude of discriminant channel, for each individual trial.

It is imperative that the training data set is independent from the data set that is to be decoded in order to avoid “double dipping”^29^. Therefore, we adopted a leave-one-out approach whenever a trial was decoded that belonged to one of the classes on which the decoder was trained. Note that no such procedure is required when the decoded data set stems from a class different from those used in training, for instance when generalizing across conditions or across blocks.

To facilitate a neurophysiological interpretation of the decoded signals, we derived a spatial projection of the discriminant channel. This spatial projection displays the coupling between the sensors and the discriminant channel and as such gives insight into which sensors contribute to the discrimination. The spatial projection **a** is given by^30^:


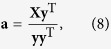


and this was subsequently fed into the source localization (see *Source localization*).

The key aspect of the current study is that we used a functional localizer in order to extract the neural signature of sensory-specific processes, and subsequently traced this signature during perceptual decision making. This was done by training the decoders on the data from the sensory processing blocks and subsequently applying these decoders to the data from the perceptual decision making blocks. That is, the decoders were generalized across blocks. In addition to this generalization, we also trained and decoded within both the sensory processing and the perceptual decision making blocks separately. Within the sensory processing blocks, the decoders were trained to discriminate between noise trials and grating trials. Within the perceptual decision making blocks, the decoders were trained to discriminate between CRs and hits. This contrast was chosen, because these conditions correspond to accurate perceptual decision making and are therefore commonly used as a baseline to which inaccurate perceptual decisions (i.e. FAs and misses) are compared. These decoders were used to decode both CRs and hits themselves (using the leave-one-out approach), as well as the FAs and misses.

Finally, we implemented the temporal generalization method to elucidate the temporal organization of the neural processing stages that underlie sensory processing and perceptual decision making^31^. When training a decoder on any specific time point, we simultaneously applied this decoder to all other time points. After averaging the obtained discriminant channel amplitude over trials, this results in a (training time) × (decoding time) matrix per condition. Comparing these matrices for two specific conditions, by subtracting one from the other to obtain a difference matrix, provides insight into how discriminability between these conditions generalizes across time. For instance, a row in such a difference matrix, corresponding to some time point *t*_train_, describes how well the two conditions can be discriminated over time on the basis of a decoder that is specifically trained, i.e. is optimally discriminative, at *t*_train_. Conversely, a column, corresponding to some time point *t*_decode_, gives insight into how well the two conditions can be discriminated at time point *t*_decode_ on the basis of the decoders trained on all other time points. As another example, consider the temporal generalization matrices obtained by generalizing from sensory processing to perceptual decision making, and consider in particular the average temporal generalization matrices for hits and CRs. Then, the difference between these matrices at the entry corresponding to some training time *t*_train_ and decoding time *t*_decode_ is a measure of how well hits can be discriminated from CRs at *t*_decode_, on the basis of the weights that was maximally discriminative between noise and grating trials at *t*_train_. The rationale behind the temporal generalization method is that the neural pattern identified by the decoder to be discriminative corresponds to some underlying neural process, and this pattern should be generated whenever that neural process is active. Thus, by testing for the presence of a particular neural pattern, one obtains an activity time course of the corresponding neural process. Note that the temporal generalization method described here is not to be confused with the between-block generalization described above.

### Statistical testing

Statistical analyses were performed on subject-level temporal generalization matrices, averaged across trials. Contrasts between conditions were tested for statistical significance using permutation tests in conjunction with cluster-based correction for multiple comparisons^32^. Specifically, univariate paired t-tests were calculated for the entire matrix. Elements that passed a threshold value corresponding to a p-value of 0.05 (two-tailed) were marked, and neighboring marked elements were collected into separate negative and positive clusters. No specific constraint was set on the minimum number of marked elements in order to be considered a cluster - that is, the minimum number of required neighbors is 1. Elements were considered neighbors if they were directly adjacent, either cardinally or diagonally. Finally, the t-values within each cluster were summed and rectified, and these values were fed into the permutation framework as the test-statistic. Consequently, all tests were two-tailed. A cluster was considered significant when it’s p-value was below 0.05. The number of permutations per contrast was 10000.

### Source localization

To substantiate the interpretation of the discriminant channels, we performed source localization on its spatial projection over time (see *Decoding analysis*). We did not *a priori* specify regions of interest and therefore made use of the minimum-norm estimation technique^33^ (as implemented in FieldTrip), rather than beamformers, because the former is the preferred technique when estimating distributed event-related source activity^34^. Single-trial covariance estimates were calculated from the data during the baseline interval of −200 to 0 ms relative to stimulus-onset. These single-trial estimates were averaged within condition and subsequently averaged over conditions. Our source model included 8196 source locations, organized along a cortical mesh based on a template brain provided by FieldTrip. The source model was aligned with each subject’s individual head position within the MEG system as determined by the three fiducial coils (see *MEG recording and preprocessing*). The covariance matrix was regularized, and the leadfields were depth-normalized and prewhitened prior to the source estimation.

The source estimation resulted in a dipole moment for each source location, over time. This dipole moment is the change in orientation and amplitude that occurs with a unit change in the discriminant channel. Thus, if a particular source does not contribute to the discriminant channel, then the dipole moment for this location has amplitude zero. We therefore reduced the dipole moments to scalar values by taking the length of the vectors, resulting in a cortical map that represents each location’s contribution to the discriminant component. However, as taking the length of a vector always results in a positive value, noise does not cancel out and therefore results in a positive bias. This bias is not uniform across source locations and as such interferes with the interpretability of the source localization. To counter this problem, we applied a permutation procedure to quantify the bias per source location and used this to normalize the source activity. Specifically, per subject and per time point of interest, trial labels were shuffled and subsequently fed into the decoding analysis and source estimation. This procedure was repeated 10000 times, resulting in a distribution of the noise for each source location. Then, noise-normalized activity at a source location is obtained by normalizing the observed activity according to the noise-distribution in that location:


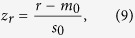


where *r* is the observed source activity and *m*_0_ and *s*_0_ are the mean and the standard deviation, respectively, of the noise distribution. Finally, these noise-normalized activity maps were averaged across subjects.

## Results

### Behavioral results

During the perceptual decision task, subjects reported perceiving the grating when it was present on 71% of trials (SD = 11%, Fig. 1C), and falsely reported perceiving it when it was not present on 14% of trials (SD = 10%), leading to a sensitivity *d’* of 1.8 (SD = 0.56) and a criterion *c* of 0.3 (SD = 0.37), indicating a slightly conservative decision bias. The average noise level across subjects, obtained from the staircase, was 95.5% (SD = 0.5%).

In the sensory processing blocks, subjects detected the oddball trials in 86% (SD = 9.5%) of the cases, while falsely reporting an oddball in only 0.3% (SD = 0.3%) of the non-oddball trials. This above-chance, yet imperfect performance verified that subjects adhered to the instructions and were well able to do the task, while also showing that the task was sufficiently difficult to require attentional engagement.

### Identifying sensory-specific neural processing

We first investigated whether we could reliably extract a neural signature that is specific to sensory processing. To this end, we focused on the neural responses during the sensory processing blocks. In these blocks, subjects were presented with noise stimuli, half of which contained an embedded grating. As these stimuli were not relevant for the task at hand,neural activity during these blocks is thus specific to sensory processing, uncontaminated by decision processes. Moreover, attention was drawn away from the stimuli towards a task at fixation, thereby minimizing potential effects of attentional modulation on the sensory processing. Although there is the theoretical possibility that the stimuli nevertheless drew attention in an automatic fashion, this bottom-up effect is presumably much weaker than the voluntary attentional engagement during perceptual decision making, and therefore most likely does not pose a problem for the present study.

**Figure 2:**
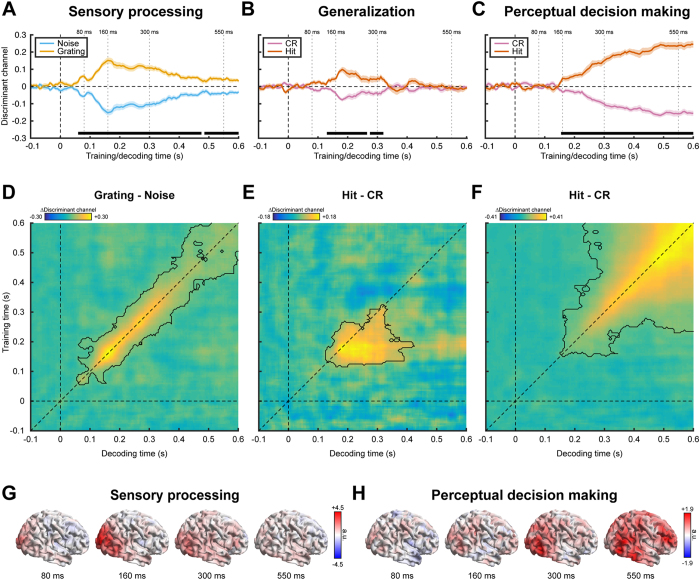
Decoding results for training and decoding within the functional localizer (A,D), for generalization from the functional localizer to correct perceptual decisions (B,E) and for training and decoding within correct perceptual decisions (C,F). (**A**–**C**) Average discriminant channel activity for noise and grating trials (**A**) and CRs and hits (**B**,**C**), at matched training and decoding time. The horizontal black bars mark time points that belong to a significant cluster as outlined in (**D**–**F**). Shaded areas depict the SEM. (**D**–**F**) Temporal generalization matrices. Note that the diagonals are identical to the differences between the curves in (**A**–**C**) and note that the color scales are variable across figure for optimal visualization. Significant clusters are demarcated by the contours. (**G**,**H**) Source level contributions to the discriminant channel trained on the functional localizer (**G**) and on perceptual decision making (**H**), at four specific time points.

We applied our decoding analysis to these data and extracted a discriminant channel that is maximally discriminative between noise and grating trials (Fig. 2A; see Supplementary Figures S1 and S2 for the same results expressed as classification accuracy and Cohen’s d, respectively). The results show that these two conditions can be reliably discriminated on the basis of the MEG recordings as evidenced by a significant cluster (p < 0.001) that extends from 60 ms post stimulus onset throughout the rest of the segment. The discriminability peaks at 80 ms and 160 ms, after which it gradually decays back toward baseline.

Next, we calculated the temporal generalization matrix of the discriminant channel to probe the temporal organization of the neural processing stages underlying sensory processing. This matrix contains the activity of the discriminant channel over time, while trained on all other time points. That means that the temporal generalization matrix provides insight into whether the conditions can be discriminated at some time point on the basis of the weights that are maximally discriminative at some other time point. The results show that, during sensory processing, the temporal generalization profile is largely located around the diagonal (Fig. 2D), indicating that a given discriminant channel only generalizes to temporally proximate neural signals. This is known as a “chain” profile^31^ and suggests that distinct, sequential neural processes are involved in the encoding of gratings versus noise. In order to interpret what these neural processes are, we assessed the source-level projection of the discriminant channel (Fig. 2G). This projection depicts the contribution of neural sources to the discriminant channel, and thus provides insight into the brain areas involved in the encoding of the stimuli. The results show that contributions stem primarily from occipital areas at both discrimination peaks, but especially prominently for the later one.

To summarize, we found that we could reliably extract a neural signature over time that is characteristic of sensory processing, in absence of decision making. This neural signature is evident throughout most of the trial and peaks at 80 and 160 ms. It encapsulates distinct, sequential neural sources over time, and these are located primarily in occipital cortex, consistent with sensory processing.

### Tracing sensory-specific processing during perceptual decision making

We proceeded to trace the sensory-specific neural signature while subjects were engaged in perceptual decision making. For this, we trained the decoders on the sensory processing blocks, serving as a functional localizer, to discriminate between noise and gratings and subsequently applied these decoders to the neural signals obtained during perceptual decision making.

We first focused on the trials in which subjects correctly reported the presence (hits) or absence (CRs) of a grating and asked whether these categories can be reliably distinguished on the basis of sensory processing. Figure 2B depicts the discriminant channel activity for these two conditions. We found a significant cluster (p = 0.006), indicating that these categories are indeed different in terms of sensory processes. Interestingly, this cluster extended from 130 to 320 ms, whereas no differences between CRs and hits were found for earlier or later time points. In addition, the cluster exhibited a relative late onset as compared to the early onset of discriminability in the sensory processing blocks. This inability to discriminate between CRs and hits before 130 ms and after 320 ms is likely due to the threshold-visibility of the gratings.

Sensory processing during perceptual decision making was found to be qualitatively different from during the functional localizer, as revealed by the temporal generalization matrix (Fig. 2E). While discriminability between CRs and hits is significant along the diagonal, i.e. when training and decoding times are matched, a substantial portion of the temporal generalization profile is found below the diagonal. This elongated shape, known as a “sustained” profile^31^, indicates that CRs and hits can be differentiated throughout an extended period of time, ranging from approximately 130 up to 400 ms, using the weights that discriminated between gratings and noise trials during the shorter interval of approximately 130 to 250 ms. In other words, the sensory representation of the stimulus, as defined during this relatively early period, is maintained over time.

**Figure 3:**
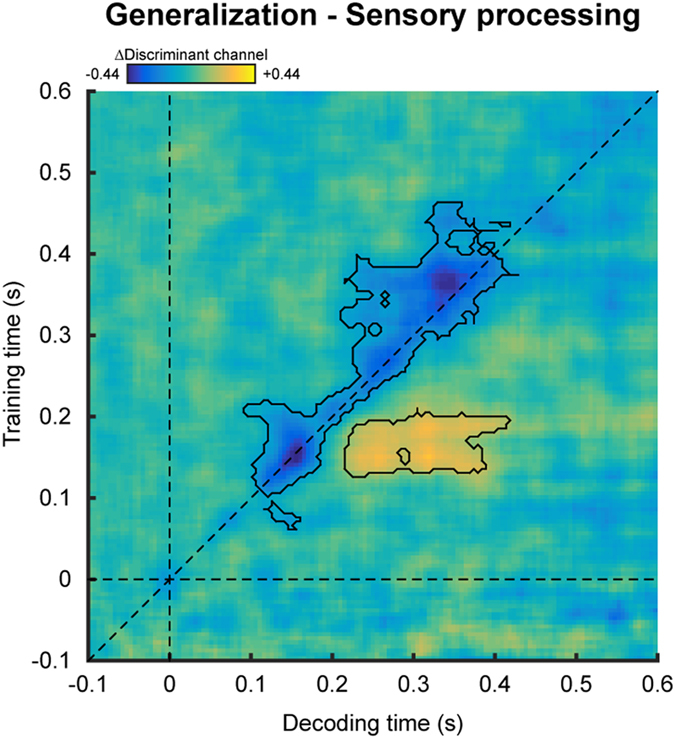
Comparison of the temporal generalization matrices depicting sensory processing during perceptual decision making (Fig. 2E**) and during unattended sensory processing (**Fig. 2D). Significant clusters are demarcated by the contours.

Direct comparison of the between-task generalization (Fig. 2E) to the temporal generalization matrix depicting sensory processing only (Fig. 2D) indeed revealed a significant positive cluster (p = 0.04) that extended from approximately 200 to 400 ms decoding time and 130 to 200 ms training time (Fig. 3). In addition, a significant negative cluster (p = 0.003) was found around the diagonal that extended from approximately 100 ms to 400 ms, showing that the diagonal decoding performance is worse during perceptual decision making than during the functional localizer. This difference is most likely due to the increased stimulus noise level during the perceptual decision making task, as compared to the localizer. In summary, these results suggest that although the overall sensory representation was weaker during perceptual decision making, the early part of this representation was stabilized and kept online in the visual system, but only when attended and/or required for a subsequent decision.

### Tracing the neural signatures of correct perceptual decisions

We also examined whether we could discriminate between the different stimulus/response-categories within the perceptual decision-making task, by performing an analysis akin to more conventional approaches, in which we did not make use of the sensory processing blocks as a functional localizer but looked at the data obtained during perceptual decision making only.

We trained the decoders to discriminate between CRs and hits and calculated the average amplitude of the discriminant channel for these categories. We found that neural processes associated with these categories begin to diverge at 155 ms and, contrary to the generalization analysis in which we decoded sensory-specific processing only, this difference remained significant (p < 0.001) throughout the rest of the trial (Fig. 2C). As CRs differ from hits not only in terms of the presented stimulus, but also with respect to the behavioral decision, the later activity differences likely reflect differences in decision-related activity - activity to which the decoders trained on the sensory processing blocks were blind.

The interpretation of the later activity as representing processes distinct from sensory encoding is corroborated by the temporal generalization matrix. In addition to the rising discriminability along the diagonal, we observed long-lasting sustained activity throughout the interval of approximately 250 ms post-stimulus until the end of the trial, meaning that CRs could be differentiated from hits during this interval using the weights obtained from any other time poi Direct comparison of the between-task generalization nt in this same interval. This “sustained” profile^31^, that can be observed in addition to a chain profile along the diagonal, shows that at least some of the neural sources remain active throughout the rest of the trial. Earlier work in monkeys demonstrated that neurons encoding for the monkey’s decision remain active in a sustained manner when the behavioral report of the decision is postponed over a delay period^35^. Thus, our results are consistent with the interpretation that the later activity reflects decision-related processing. Conversely, earlier discriminant channel activity prior to this sustained activity, up to approximately 250 ms, does not show this widespread temporal generalization, suggesting that different processes are at work in this time window, possibly transient sensory processes.

Finally, to substantiate these interpretations, we asked which brain areas contribute to the discrimination between CRs and hits. At both 160 ms and 300 ms, discriminant activity is observed selectively over occipital cortex. Interestingly, and in line with the interpretation that the later signal reflects a decision process, discriminant activity at 550 ms is much more widespread and encompasses parietal and frontal cortices, which are often implicated in decision making^1^,^2^. It is also noteworthy that the contribution in occipital regions to the discriminant channel increases from 160 ms to 300 ms when a perceptual decision is made, whereas it decreases between these two time points in the functional localizer blocks. Indeed, this is in agreement with the observation that the sensory representation in the brain is enhanced and maintained over time when required for the task at hand.

### Sensory processing during perceptual decision errors

**Figure 4:**
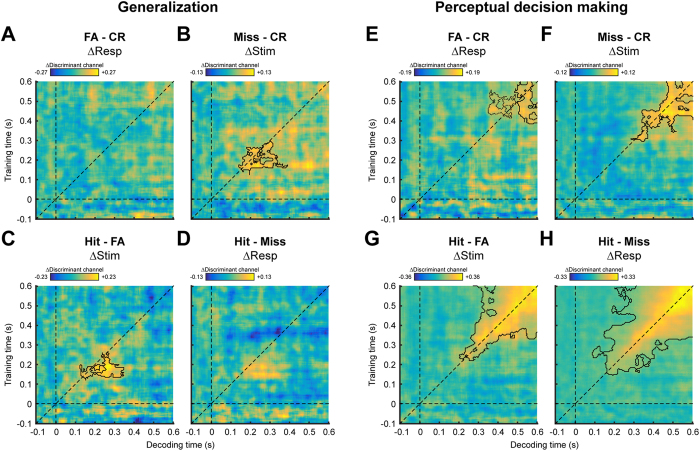
Temporal generalization matrices comparing correct to incorrect perceptual decisions, for generalization from the functional localizer to perceptual decision making (A–D) and for training and decoding within perceptual decision making (E–H). The subtitles emphasize the factor on which the contrasts vary. Note that the color scales are variable across figure for optimal visualization. Significant clusters are demarcated by the contours.

So far we focused exclusively on correct perceptual decisions - i.e., CRs and hits, which differ on both stimulus and decision dimensions. We extended our previous between-block generalization analysis to the incorrect perceptual decisions and decoded FAs and misses, in addition to CRs and hits, using decoders that were trained on neural signals obtained during sensory processing (Fig. 4A–D; see Supplementary Figuers S3 and S4 for the same results expressed as classification proportions and Cohen’s d, respectively). These categories were then compared to CRs and hits to assess how incorrect perceptual decisions deviate from correct ones with respect to sensory processing. This is interesting, because there has been considerable debate in the literature whether perceptual decision errors stem from faulty sensory encoding^12^,^36^,^37^ or are instead the result of noise in supramodal decision making^13^,^15^,^38^. Our approach using the between-task generalization is in a position to resolve this question, as it was specifically designed to probe sensory processing only.

We found that misses could be reliably discriminated from CRs (p = 0.007, Fig. 4B), and hits from FAs (p = 0.016, Fig. 4C). As was the case for the comparison of CRs to hits described above (Fig. 2E), the time period during which a discriminative signal was present ranged from approximately 150 ms to 300 ms. Furthermore, the temporal generalization matrix of these comparisons also displayed an elongated, below-diagonal profile. In contrast, no significant differentiation was obtained between the contrasts of FA versus CR (Fig. 4A) and hits versus misses (Fig. 4D). Although a trend may be discerned in the latter contrast, this did not constitute a significant cluster. In short, the decoders were only able to discriminate between conditions that are different with respect to stimulus, but not between conditions that differ on decision. Therefore, given that these decoders were designed to target sensory processing only, these results show that the encoded sensory information accurately reflects the physical stimulus, even when an incorrect decision follows. These findings suggest that, in the current study, perceptual decision errors stemmed mainly from later decision-related processes, rather than from faulty sensory encoding.

We also subjected the perceptual decision errors to the within-task approach in which we trained on CRs and hits within perceptual decision making and used these weights to decode the perceptual decision errors (Fig. 4E–H). We found that FAs could be reliably separated from CRs during a relatively late time period (Fig. 4E; solid cluster: p = 0.025, dotted cluster: p = 0.05), but not during an earlier time period, consistent with the interpretation that the discriminant channel in the late period reflects decision processes. Interestingly, the situation is different when comparing hits to misses. Although these categories are also expected to differ only during the later decision period, we found that the significant cluster extended to earlier time points (p < 0.001, Fig. 4H). More discrepancies were found when comparing misses to CRs (Fig. 4F) and hits to FAs (Fig. 4G). As these categories differ only in terms of presented stimulus, they are expected to be discriminable solely during the early sensory period. This is however not what we found. Instead, these comparisons revealed significant clusters in late time windows (CR versus miss: p = 0.013; FA versus hit: p < 0.001), but not in early ones, suggesting that these categories differ in decision-related neural processing, despite identical behavioral response. These discrepancies highlight the difficulties in disentangling sensory from decision processes when only considering neural data obtained during perceptual decision making, without making use of a functional localizer, and we elaborate on this issue in the *Discussion*.

Finally, to consider the possibility that these discrepancies may have arisen from the fact that we trained on CRs versus hits instead of noise versus grating (as in the localizer), we conducted an additional analysis in which we trained on stimulus only, irrespective of the response, and used this decoder to compare the four categories (Supplementary Fig. S5). These analyses resulted in the same discrepancies mentioned above. FAs are significantly different from hits (p < 0.001), but not from CRs - in line with what one would expect, given that these decoders should be sensitive to differences in stimulus only. Again however, there were significant differences between misses and hits (p = 0.004), but not between CRs and misses. Moreover, all of these differences were confined to relatively late time windows (>±300 ms), whereas no significant differences were found before that. These results are at odds with the results obtained from the between-task generalization, where we only found effects during early time windows (approximately 130 to 320 ms), and indeed highlight the utility of using a functional localizer.

## Discussion

In the present study we sought to dissociate sensory processing from decision-related processing during perceptual decision making. We accomplished this using a novel approach where we employed a functional localizer task to identify the neural signature specific to sensory processing, and used this to trace the temporal trajectory of sensory encoding during perceptual decision making. Our results revealed a temporal dissociation between sensory- and decision-related neural activity. We found that that sensory information was encoded in neural signals during a relative early time window that extended from 130 to approximately 350 ms post stimulus, and that this encoded sensory information correctly reflected the physical stimulus even in the case of an incorrect decision. In contrast, we found a later, sustained decision-related neural process that extended from approximately 250 until at least 600 ms and become more pronounced over time, in agreement with previous reports^7^,^10^. Moreover, although gratings could be distinguished for a longer period of time in the functional localizer as compared to perceptual decision making, the sensory representation in the former was unstable and changing over time. During perceptual decision making on the other hand, the early sensory representation was stable for an extended period of time. Thus, these results show that the early sensory representation was stabilized and maintained over time when required for a decision, but not when the stimulus was unattended and task-irrelevant.

A number of previous studies have also focused on disentangling sensory and decision processes^4^,^5^,^6^,^7^,^8^,^9^,^10^. For instance, Wyart *et al.*^8^ orthogonalized these processes by randomizing the decision-relevant information of the stimulus on a trial-to-trial basis, such that it did not correlate with the raw sensory information. This led to a temporal dissociation in which sensory-related signals preceded decision-related activity, similarly to our results. However, whereas these studies relied on external manipulation of the stimulus while assuming constant internal sensory processing, we instead kept the stimulus constant and capitalized on ongoing fluctuations in perception and/or decision making in order to extract decision-related activity. This paradigm is widely employed for a variety of purposes, for instance to uncover the neural correlates of consciousness^39^,^40^,^41^ or to extract internal perceptual templates^42^,^43^. However, as explained in the *Introduction*, using behavioral report as independent factor introduces interpretational limitations, as it is an observed variable and therefore not under experimental control. One commonly used way to facilitate interpretation is to delineate neural activity in the spatial domain (e.g. ref. 15). For instance, activity in motion sensitive area MT is parametrically modulated by visual motion strength^44^,^45^ and may therefore be defined as encoding for sensory evidence. Similarly, activity in parietal areas has been found to exhibit characteristics of integration toward a decision boundary, with a rate proportional to the signal strength^7^,^10^,^35^, and may therefore be defined as encoding for the decision process. However, dissociation on the basis of spatial location may be fallacious, because the activity of sensory neurons does not only reflect the stimulus, but can also reflect decision processes due to interactions between cognitive processes and sensory neurons^46^,^47^,^48^. Therefore, merely recording from sensory areas may be insufficient to disentangle sensory processing from decision-related activity. In fact, these results point toward a more general, conceptual problem. Namely, that perceptual decisions are not the result of a sequential processing pipeline, but rather stem from complex, reciprocal interactions within a large network of areas^49^. It is therefore conceptually challenging to unambiguously identify the neural signals underlying sensory processing when these signals are also used for decision making. This holds for all brain recording methods, as even the availability of superior spatial resolution, such as in single-cell recordings, does not resolve this problem.

We attempted to counter this problem by means of a separate functional localizer, which allowed us to unambiguously define the sensory-specific neural signature, in the absence of decision making or attentional modulation. The use of a functional localizer is conceptually similar to a common practice in single-cell recordings, where the tuning properties of neurons are mapped in a separate session. Our approach extends this idea to human neuroimaging, and yielded two important results. First, we were able to address the following question: how does the brain reliably integrate sensory evidence if the stimulus is available for only a very limited amount of time? According to the sequential sampling framework, the decision variable is constructed by means of sequential sampling of the sensory evidence over time^1^,^3^. This makes sense in the case where a stimulus is presented for a prolonged period of time, such as in the commonly used random-dot motion stimulus. However, it is not intuitive how sequential sampling should proceed after a brief stimulus has disappeared. Computational modeling work^50^ suggests that sensory evidence has to remain available to the accumulator after stimulus offset in order to fit the observed data. Here we present a direct experimental demonstration of this assumption. Our results show that the sensory representation is maintained over time during perceptual decision making, but not when the stimulus is viewed while attention is directed away from it. Thus, it appears that the brain actively stabilizes the sensory representation, presumably by means of top-down mechanisms such as attention^13^ or working memory^50^,^51^, when it is required for a subsequent decision.

Second, when comparing incorrect perceptual decisions (FAs and misses) to correct decisions (CRs and hits), we observed discrepancies between the decoded neural signals in the case where the decoders were trained on the perceptual decision making data and the case where the decoders were trained on the functional localizer. In the latter, we only found differences between the stimulus/response-categories that varied in terms of stimulus (CR versus miss; FA versus hit) and not between the categories in which physical stimulation was identical (CR versus FA; miss versus hit). Indeed, these differences were exclusive to a relatively early time window, as would be expected given that this early time period reflects sensory processing. However, different results were obtained when the decoders were trained on the neural signals recorded during correct perceptual decisions. In this case we did not observe early differences between CRs and misses, but we did find early differences between misses and hits. Thus, these results suggest that early neural activity reflects the eventual decision, rather than physical stimulation.

This paradox can be resolved by considering the data sets on which the decoders were trained in each case. In the case of the functional localizer, the conditions in the training data (noise versus grating) varied only with respect to physical stimulation. When training on the perceptual decision making data however, the conditions in the training data (CR and hit) diverged not only with respect to physical stimulation, but also with respect to behavioral decision. As behavioral decision is an observed variable, it is not under experimental control and its effect is therefore susceptible to confounding variables. One possible instantiation of such a confound would be trial-to-trial fluctuations in attention^13^,^22^,^52^, that modulate the likelihood that an encoded grating is successfully transferred into the decision process. This would explain the discrepancy, because hits would be inherently accompanied with a different ongoing attentional state relative to misses, for else they would have been classified as a miss. No such bias would be present for the CRs, because no grating is encoded in these trials. Together with the threshold-visibility of the grating, we therefore suggest that the decoders that were discriminative between CRs and hits at early time points were primarily sensitive to these fluctuations, rather than to physical stimulation. As these fluctuations have an impact on the eventual behavioral decision, this provides an explanation for the decision-related activity at early time points when training on CRs versus hits. Indeed, it has recently been proposed that decision-related activity in sensory neurons may result from ongoing fluctuations in higher-level expectations about the upcoming stimulus, whose activity is projected back to lower-level sensory areas^46^,^53^,^54^.

Finally, although we interpreted the later discriminant channel activity as reflecting the decision process, we nevertheless found differences in this time window between categories that are identical in terms of behavioral response. One explanation for this discrepancy may be that the integration of sensory evidence during incorrect decisions follows a deviating trajectory as compared to correct decisions^3^,^17^. A second explanation is that subjective confidence in a perceptual decision is likely to be different between correct and incorrect decisions and, given that subjective confidence is manifested in electroencephalography signals^17^, may therefore have given risen to the observed differential activity.

In conclusion, we presented a an empirical dissociation between sensory processes and decision-related processes during human perceptual decision making. Our results are largely consistent with previous findings, but also provide new insights into the mechanisms underlying perceptual decision making. Our results suggest that a sensory representation is maintained when required for the task at hand and/or attended. Importantly, we also found that sensory processes accurately encode the physical stimulus during perceptual decision errors. We believe that our approach, as well as the insights obtained with it, make an important contribution to various fields of study, including that of perceptual decision making, the neural correlates of consciousness and visual cognition.

## Additional Information

**How to cite this article**: Mostert, P. *et al.* Dissociating sensory from decision processes in human perceptual decision making. *Sci. Rep.* **5**, 18253; doi: 10.1038/srep18253 (2015).

## Supplementary Material

Supplementary Information
