# Supplementary material for: Dissociating sensory from decision processes in human perceptual decision making
Source: Sci Rep. 2015 Dec 15;5:18253. doi: 10.1038/srep18253 (PMC4678878; doi:10.1038/srep18253)
Supplement: Supplementary Information [file srep18253-s1.pdf]

Dissociating sensory from decision processes in human perceptual decision making

Pim Mostert[1,*] , Peter Kok[1], Floris P. de Lange[1]

[1] Radboud University, Donders Institute for Brain, Cognition and Behaviour, 6500 HB, Nijmegen, The Netherlands

[*] Corresponding author:

Pim Mostert

Donders Institute for Brain, Cognition and Behaviour

Radboud University Nijmegen

P.O. Box 9101, 6500 HB Nijmegen, The Netherlands

Voice: 31 24 36 10889

Fax: 31 24 36 10989

E-mail: p.mostert@donders.ru.nl

Supplementary Information

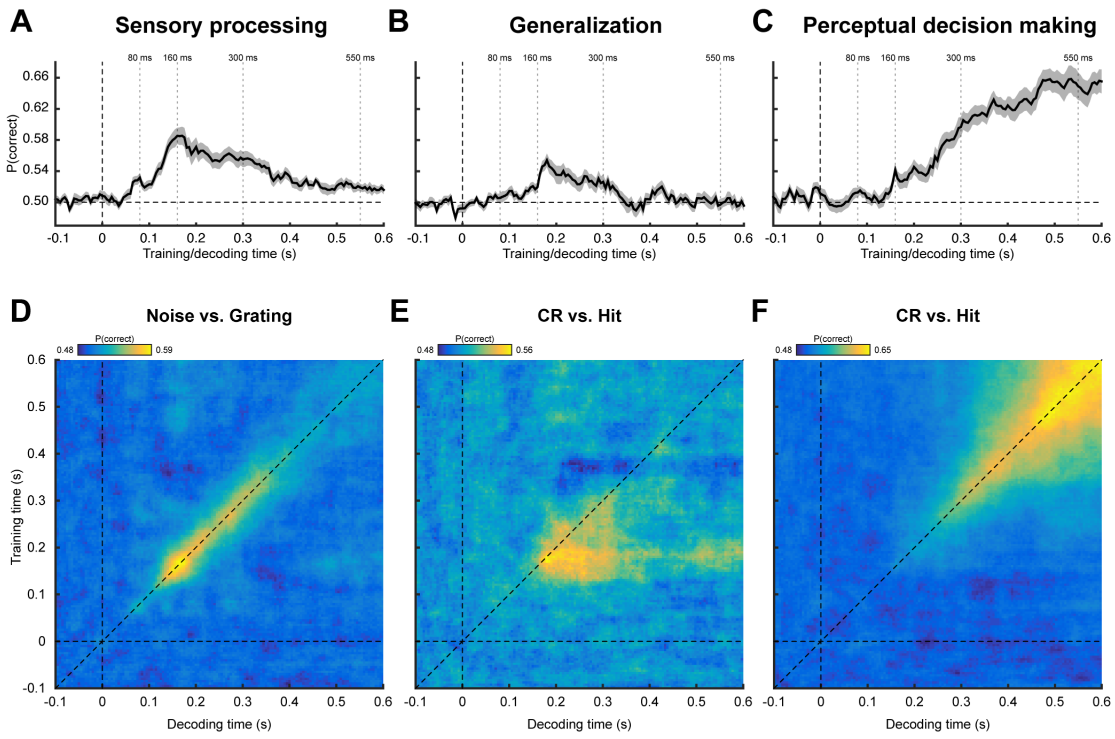

Supplementary Figure S1. The same results as presented in Figure 2A-F, but instead expressed as classification accuracy. For the between-task generalization, hits and CRs were considered correct when classified as gratings and noise, respectively. No statistical tests were conducted on these results.

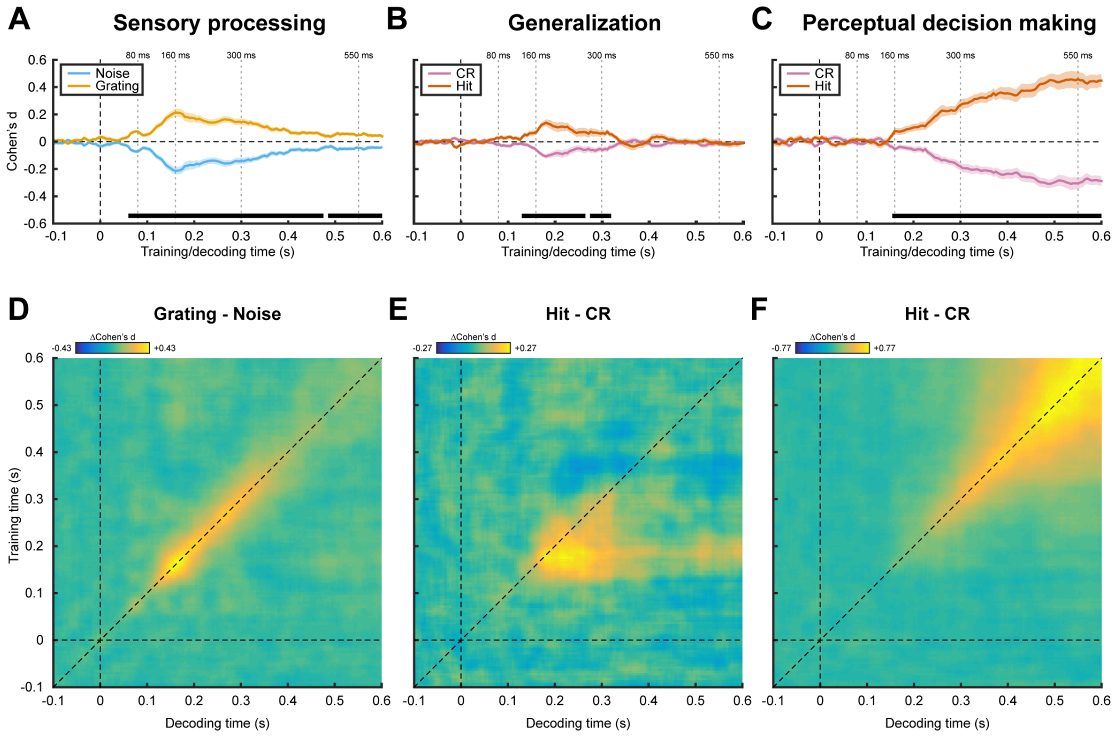

Supplementary Figure S2. The same results as presented in Figure 2A-F, but instead expressed as Cohen's d. No statistical tests were conducted on these results.

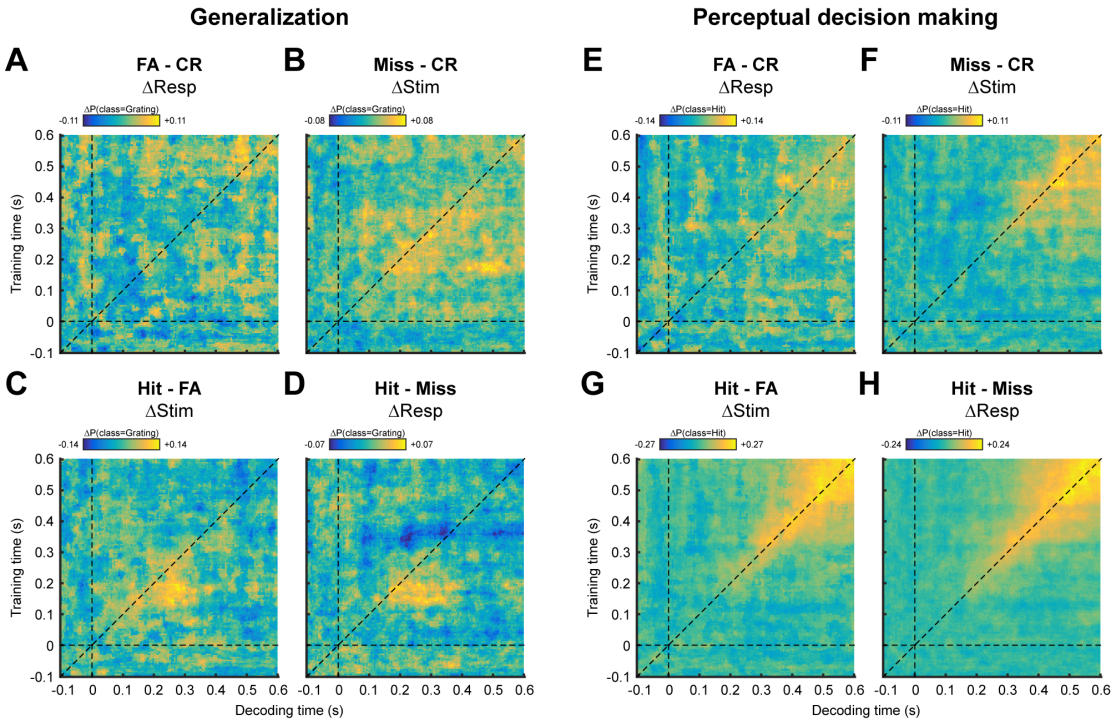

Supplementary Figure S3. The same results as presented in Figure 4, but instead expressed as the proportion of trials within each of the four stimulus/response-categories that were classified as grating (***A-D***) or as hit (***E-H***). No statistical tests were conducted on these results.

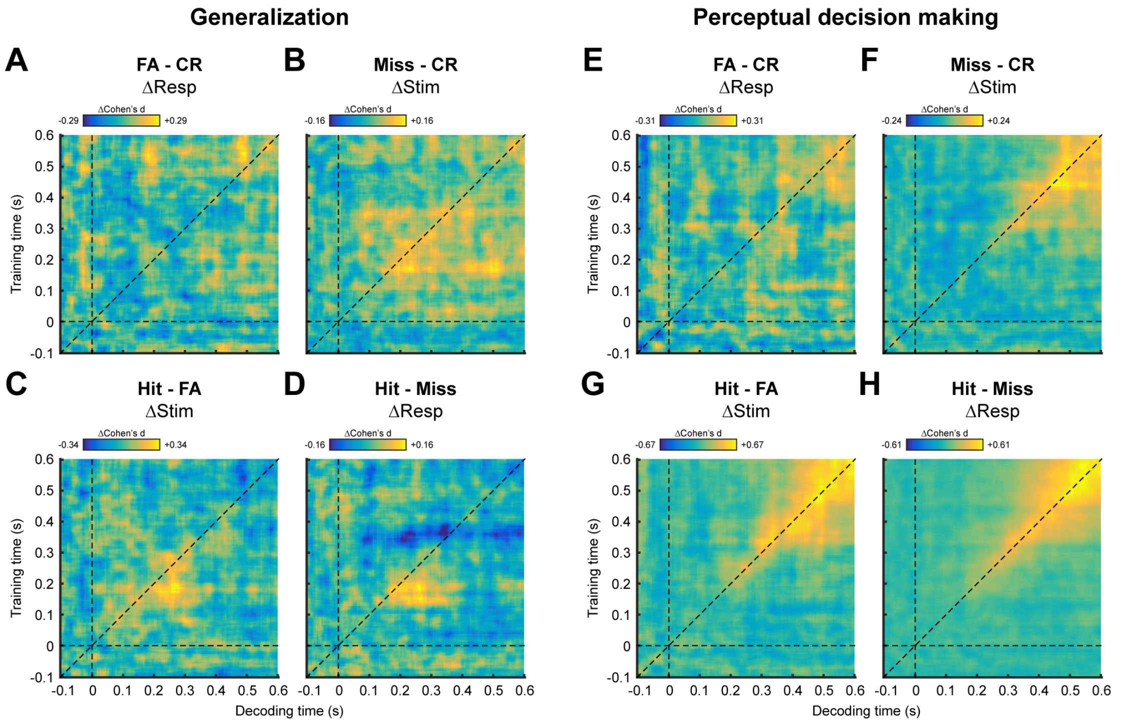

Supplementary Figure S4. The same results as presented in Figure 4, but instead expressed as Cohen's d. No statistical tests were conducted on these results.

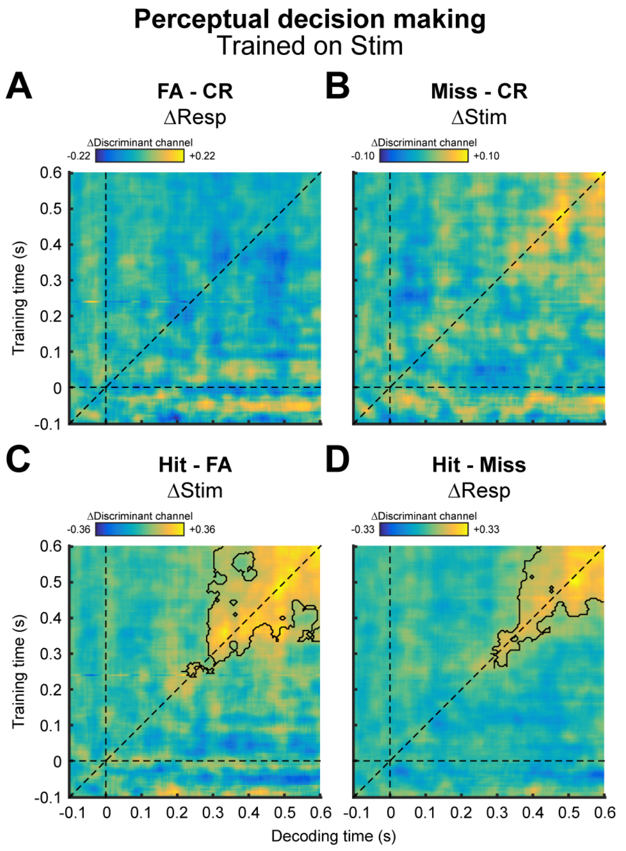

Supplementary Figure S5. The results of an analysis akin to Figure 4, but instead the decoders were trained to discriminate between grating present versus absent, irrespective of the subject's decision (as opposed to training on CRs versus hits). However, as the numbers of trials are unequal across the four categories, the stimulus presence is correlated to decision. To counter this, we trained on the unweighted average of CRs and FAs (i.e. stimulus absent) versus the unweighted average of misses and hits (i.e. stimulus present). Specifically, in Eq. 5 (see *Methods*), this corresponds to $\hat{\mu}_1 = \frac{1}{2}(\hat{\mu}_{CR} + \hat{\mu}_{FA})$ and $\hat{\mu}_2 = \frac{1}{2}(\hat{\mu}_{Miss} + \hat{\mu}_{Hit})$. Similarly, the common covariance matrix was calculated as the unweighted average of the four individual covarance matrices within each category.
